# Impact of low serum albumin levels on cardiovascular deaths in cancer survivors with heart failure

**DOI:** 10.1007/s00380-025-02652-1

**Published:** 2026-01-21

**Authors:** Ken Watanabe, Azusa Kuroyanagi, Tomonori Aono, Satoshi Aita, Hiromasa Hasegawa, Hyuma Daidoji, Katsuaki Takahashi, Yoshiaki Tamada, Akio Fukui

**Affiliations:** 1 Department of Cardiology, Pulmonology, and Nephrology, Yamagata University School of Medicine, 2-2-2 Iida-Nishi, Yamagata, 990-9585 Japan; 2 Department of Cardiovascular Medicine, Yamagata Prefectural Central Hospital, Yamagata, Japan

**Keywords:** Cancer survivors, Heart failure, Cardiovascular death, Serum albumin

## Abstract

Advancements in cancer treatment have iproved the long-term prognosis, leading to an increasing number of cancer survivors with coexisting heart failure (HF). Cardiovascular (CV) death is one of the major causes of death among cancer survivors with HF, highlighting the crucial need for risk stratification for CV death to improve their long-term prognosis. However, the specific risk factors for CV death in cancer survivors with HF are not fully understood. We enrolled 485 cancer survivors who were admitted to our hospital for treatment of HF between 2011 and 2023. All patients were prospectively followed up for a median period of 545 days with the endpoint of all-cause death. During the follow-up period, there were 107 CV deaths, which accounted for more than half of the total deaths and were four times higher than cancer deaths. Multivariate Fine-Gray analysis showed that low serum albumin levels independently predicted CV death after adjustment for confounding factors (sub-distribution hazard ratio [sHR], 0.740 per 1-SD increase; 95% confidence interval [CI], 0.580–0.946; *P* = 0.016). When using the albumin cut-off value of 3.7 g/dL for CV death, the patients with low albumin levels had significantly higher risk for CV death (sHR, 0.557; 95% CI, 0.376–0.825; *P* = 0.004). Low serum albumin levels were identified as an independent predictor of CV death in cancer survivors with HF. Serum albumin levels may be a useful biomarker for improving the prognosis of this high-risk population.

## Introduction

With improvements in cancer detection and therapy, the number of cancer survivors continues to rise [1]. One of the most important complications among cancer survivors is cardiovascular (CV) disease, particularly heart failure (HF) [2]. The increased risk of HF in cancer survivors is multifactorial, involving cardiotoxic cancer therapies (e.g., anthracyclines, trastuzumab) and shared risk factors such as aging, hypertension, and diabetes mellitus [3, 4]. CV death, rather than cancer-related mortality, is increasingly becoming the major causes of death among long-term cancer survivors [5]. Patients with both cancer and HF may represent a particularly high-risk group with a poor prognosis, highlighting the crucial need for risk stratification for CV death to improve their long-term clinical outcomes. However, the specific risk factors for CV death in cancer survivors with HF remain inadequately elucidated.

Serum albumin is a well-established prognostic biomarker in both oncology and cardiology. Low serum albumin reflects a combination of malnutrition, systemic inflammation, and cachexia in cancer patients, and it is associated with adverse outcomes including mortality [6, 7]. Similarly, in patients with HF, hypoalbuminemia correlates with congestion, impaired nutritional reserve, and systemic inflammation, and has been reported consistently associated with adverse clinical outcomes [8]. Importantly, cancer survivors who develop HF represent a population in which cancer- and HF-related catabolic and inflammatory processes may exist and interact [9]. Albumin could capture overlapping pathophysiology that is not fully reflected by traditional HF biomarkers such as natriuretic peptides. However, it is uncertain whether serum albumin levels can independently predict CV death when considering the competing risk of cancer-related death in cancer survivors with HF. In this study, we aimed to identify independent predictors of CV death, including albumin as a candidate predictor, in this specific population.

## Materials and methods

### Study subjects

The present study enrolled 485 cancer survivors who were admitted to our hospital for the treatment of HF between 2011 and 2023. The clinical diagnosis of HF was based on the Framingham criteria, which requires the presence of either two major criteria, or one major and two minor criteria [10]. Typical major criteria (e.g., orthopnea, neck vein distension, rales, acute pulmonary edema) and minor criteria (e.g., ankle edema, dyspnea on exertion) were assessed by treating physicians. In this study, the cancer survivor was defined as an individual with a previous diagnosis of malignancy who had completed primary cancer therapy and was not undergoing active treatment for recurrent or metastatic disease at the time of HF admission. This study excluded patients who experienced acute coronary syndrome within three months preceding admission and estimated life expectancy is less than three months. Baseline clinical data were collected at the time of admission, including age, sex, medical history, comorbidities, laboratory parameters, and cancer type and treatment history. Medications were assessed at discharge. Echocardiographic data were obtained within 48 h of admission. The investigation conforms with the principles outlined in the *Declaration of Helsinki*. The study protocol was approved by the institutional ethics committee of Yamagata Prefectural Central Hospital, and all patients provided their written informed consent for study participation.

### Endpoint and follow-up period

All patients were prospectively followed up until the endpoint of all-cause death or the last follow-up visit, with a median period of 545 days (interquartile range (IQR): 112–1301 days). Mortality information was obtained from electrical medical records. Causes of death were classified as CV death, cancer-related death, or death from other causes. CV death was defined as death due to worsening HF, myocardial infarction, stroke, fatal arrhythmia, or sudden cardiac death.

### Statistical analysis

The results are expressed as the mean ± standard deviation (SD) for continuous variables, and percentages for categorical variables. Skewed values are presented as the median and IQR. We used t-tests and chi-square tests to compare continuous and categorical variables, respectively. If the data were not normally distributed, the Mann–Whitney *U*-test was employed. Differences among groups were analyzed by one-way analysis of variance, the Kruskal–Wallis test, and the chi-squared test. The cumulative incidence-time curves for time to CV death were constructed using the Gray test. The Fine-Gray model was employed to estimate sub-distribution hazard ratio (sHR) for CV death, accounting for competing risks such as cancer-related death and death from other causes. Significant variables from the univariate analysis were then entered into the multivariate analysis. The receiver-operating characteristic (ROC) curve was used to identify the optimal cut-off value of serum albumin for predicting CV death. *P*-values of < 0.05 were considered statistically significant. All statistical analyses were performed with a standard software package (JMP version 18, SAS Institute, Cary, NC, USA; EZR, Saitama Medical Center, Jichi Medical University, Shimotsuke, Japan).

## Results

### Comparison between patients with and without death

**Fig. 1:**
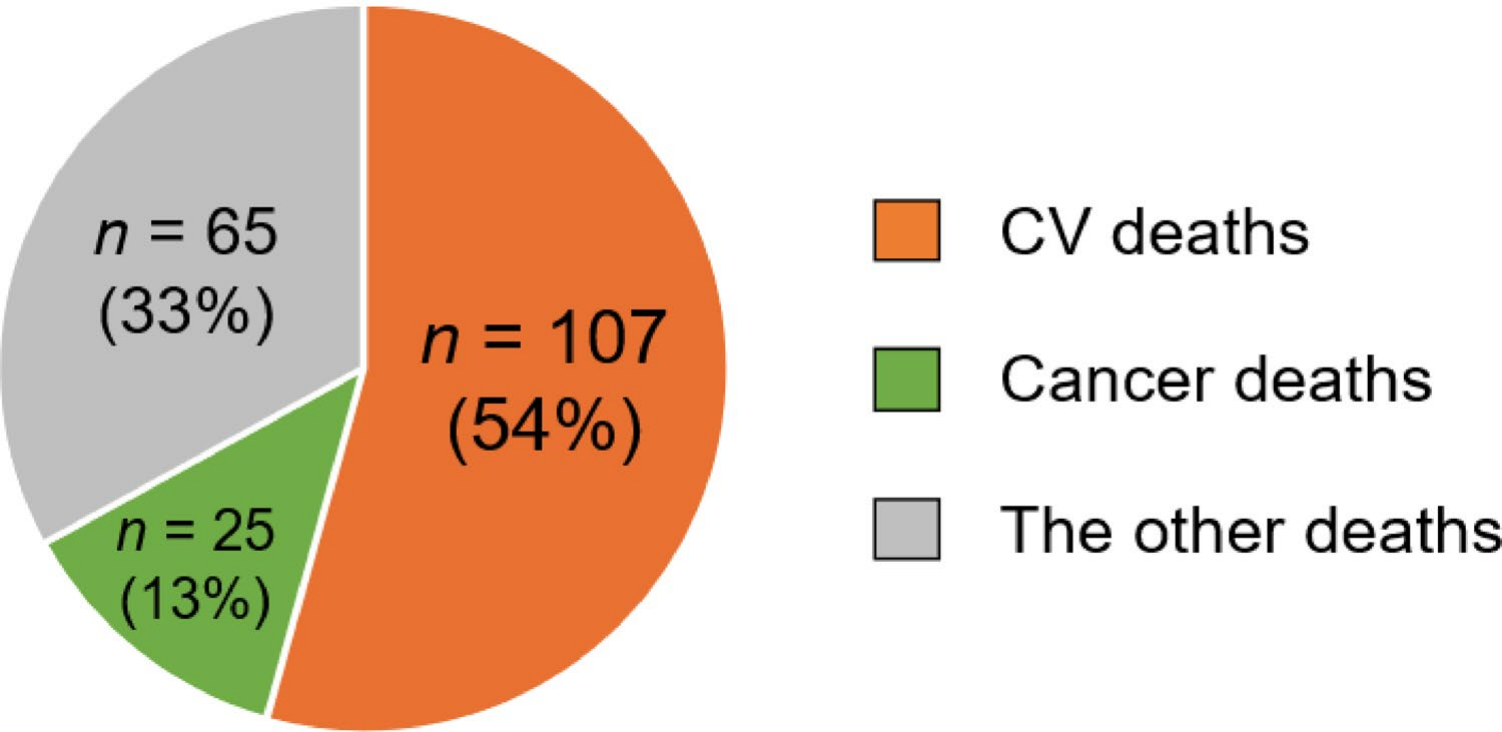
The proportion of causes of death. CV, cardiovascular

**Fig. 2:**
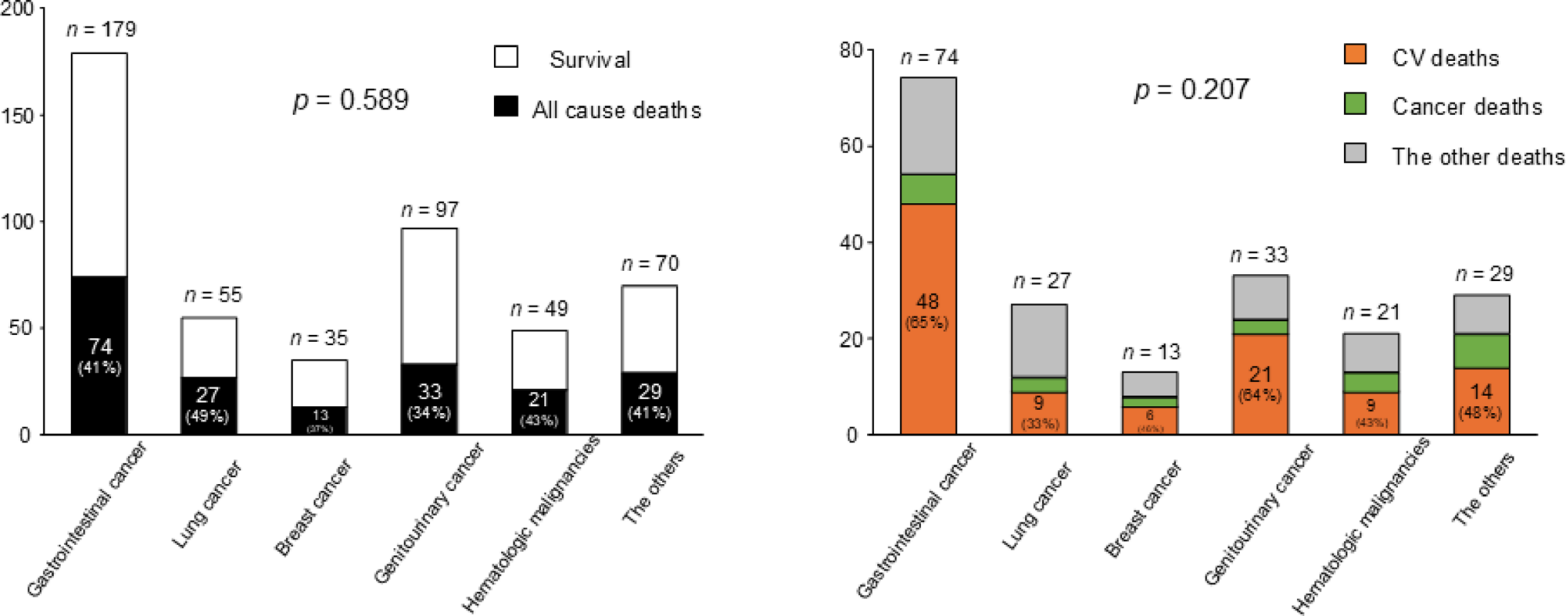
The cause-specific mortality in each cancer category. CV, cardiovascular

During the follow-up period, there were a total of 197 deaths, of which 107 patients were attributed to CV causes. CV deaths accounted for more than half of the all-cause deaths and were four times more frequent than cancer-related deaths (Fig. 1). The proportion of patients by cancer types was shown in Fig. 2. There were no significant differences in all-cause mortality rates among cancer types. Similarly, no significant differences were observed in the distribution of CV deaths, cancer-related deaths, and other causes of death across cancer types. A comparison of the baseline clinical characteristics of the patients with and without death is shown in Table 1. The patients who died were more often male, had a higher prevalence of hypertension, and had a history of prior hospitalization for HF compared with those without death. There were no significant differences in echocardiographic data and medications at discharge between the groups. The patients who died had significantly lower serum albumin levels and hemoglobin levels than those who survived.

**Table 1:** Comparison of the baseline clinical characteristics of patients with and without all-cause death

Variables	Overall *n* = 485	Death (–) *n* = 288	Death ( +) *n* = 197	*P* value
Age (years)	81 ± 9	81 ± 10	81 ± 9	0.797
Male, *n* (%)	308 (63)	168 (58)	134 (68)	0.030
BMI (kg/m^2^)	21.1 ± 3.7	21.3 ± 3.8	21.0 ± 3.7	0.375
Hypertension, *n* (%)	356 (73)	201 (70)	155 (79)	0.028
Diabetes mellitus, *n* (%)	248 (51)	145 (50)	103 (52)	0.675
Dyslipidemia, *n* (%)	123 (25)	74 (26)	49 (25)	0.838
Atrial fibrillation, *n* (%)	263 (54)	162 (56)	101 (51)	0.280
Prior hospitalization for HF, *n* (%)	254 (52)	136 (47)	118 (60)	0.005
Echocardiographic data				
LVEDD (mm)	50 ± 9	50 ± 9	50 ± 10	0.866
LVEF (%)	52 ± 17	52 ± 17	52 ± 18	0.911
LAD (mm)	45 ± 9	45 ± 8	45 ± 9	0.722
Blood examination				
Albumin (g/dL)	3.6 ± 0.5	3.6 ± 0.5	3.5 ± 0.5	0.001
eGFR (mL/min/1.73 m^2^)	46 ± 23	48 ± 22	44 ± 24	0.098
Hemoglobin (g/dL)	11.2 ± 2.4	11.4 ± 2.3	10.9 ± 2.4	0.027
BNP (pg/mL)	607 (292–1137)	534 (282–1093)	714 (319–1318)	0.151
Medications at discharge				
ACEIs and/or ARBs, *n* (%)	271 (56)	170 (59)	101 (51)	0.091
β-blockers, *n* (%)	259 (53)	164 (57)	95 (48)	0.059
Diuretics, *n* (%)	359 (74)	216 (75)	143 (73)	0.553

Data are expressed as mean ± SD, number (percentage), or median (interquartile range). ACEIs, angiotensin-converting enzyme inhibitors; AF, atrial fibrillation; ARBs, angiotensin II receptor blockers; BMI, body mass index; BNP, B-type natriuretic peptide; HF, heart failure; eGFR, estimated glomerular filtration rate; LAD, left atrial diameter; LVEDD, left ventricular end-diastolic diameter; LVEF, left ventricular ejection fraction.

Furthermore, Table 2 summarizes the clinical characteristics of patients stratified by causes of death: no death, non-CV death, and CV death. There were no significant differences among the three groups in terms of age, body mass index, medications, and prevalence of hypertension, diabetes mellitus, dyslipidemia, and atrial fibrillation. The proportion of male patients was significantly higher in the non-CV death group. The CV death group had the greatest proportion of prior hospitalization for HF and had significantly higher B-type natriuretic peptide (BNP) levels than in the other groups. Although left ventricular end-diastolic diameter, left atrial diameter, and left ventricular ejection fraction (LVEF) did not significantly differ among the groups, there was a trend toward lower LVEF in the CV death group compared to the other groups. Serum albumin levels were significantly lower in the CV and non-CV death groups compared to the no-death group, while estimated glomerular filtration rate (eGFR) and hemoglobin levels did not significantly differ among the three groups.

**Table 2:** Comparison of the clinical characteristics among three groups based on causes of death

Variables	Death (–) *n* = 288	Non-CV deaths *n* = 90	CV deaths *n* = 107	*P* value
Age (years)	81 ± 10	80 ± 9	81 ± 9	0.352
Male, *n* (%)	168 (58)	65 (72)	69 (65)	0.048
BMI (kg/m^2^)	21.3 ± 3.8	20.9 ± 3.8	21.0 ± 3.6	0.673
Hypertension, *n* (%)	201 (70)	72 (80)	83 (78)	0.083
Diabetes mellitus, *n* (%)	145 (50)	50 (56)	53 (50)	0.642
Dyslipidemia, *n* (%)	74 (26)	24 (27)	25 (23)	0.849
Atrial fibrillation, *n* (%)	162 (56)	41 (46)	60 (56)	0.189
Prior hospitalization for HF, *n* (%)	136 (47)	50 (57)	68 (64)	0.011
Echocardiographic data				
LVEDD (mm)	50 ± 9	50 ± 9	50 ± 10	0.983
LVEF (%)	52 ± 17	55 ± 17	49 ± 17	0.057
LAD (mm)	45 ± 8	44 ± 8	45 ± 9	0.753
Blood examination				
Albumin (g/dL)	3.6 ± 0.5	3.5 ± 0.6	3.5 ± 0.5	0.002
eGFR (mL/min/1.73 m^2^)	48 ± 22	46 ± 28	42 ± 21	0.150
Hemoglobin (g/dL)	11.4 ± 2.3	10.9 ± 2.4	10.9 ± 2.4	0.086
BNP (pg/mL)	534 (282–1093)	582 (281–908)	805 (336–1510)	0.034
Medications at discharge				
ACEIs and/or ARBs, *n* (%)	170 (59)	44 (49)	57 (53)	0.199
β-blockers, *n* (%)	184 (57)	43 (48)	52 (49)	0.166
Diuretics, *n* (%)	216 (75)	64 (71)	79 (74)	0.766

Data are expressed as mean ± SD, number (percentage), or median (interquartile range). ACEIs, angiotensin-converting enzyme inhibitors; AF, atrial fibrillation; ARBs, angiotensin II receptor blockers; BMI, body mass index; BNP, B-type natriuretic peptide; CV, cardiovascular; eGFR, estimated glomerular filtration rate; HF, heart failure; LAD, left atrial diameter; LVEDD, left ventricular end-diastolic diameter; LVEF, left ventricular ejection fraction

### The predictors of cardiovascular mortality in cancer survivors with heart failure

**Fig. 3:**
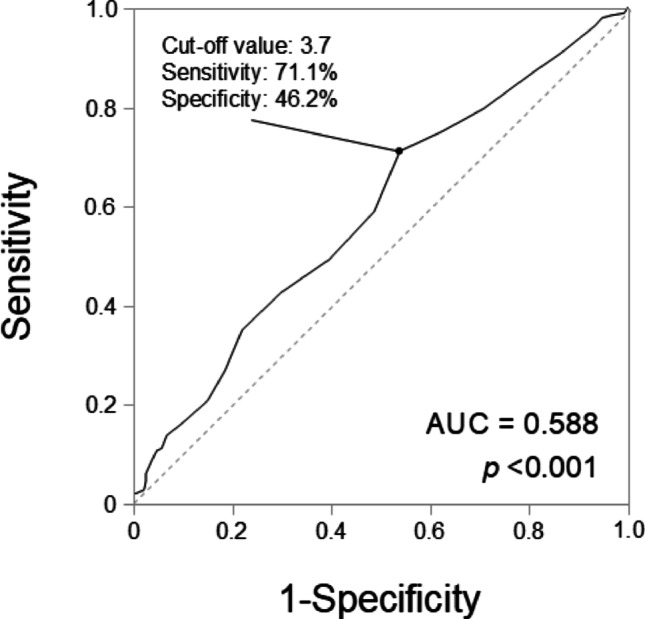
The receiver operating characteristic curve for predicting cardiovascular death

**Fig. 4:**
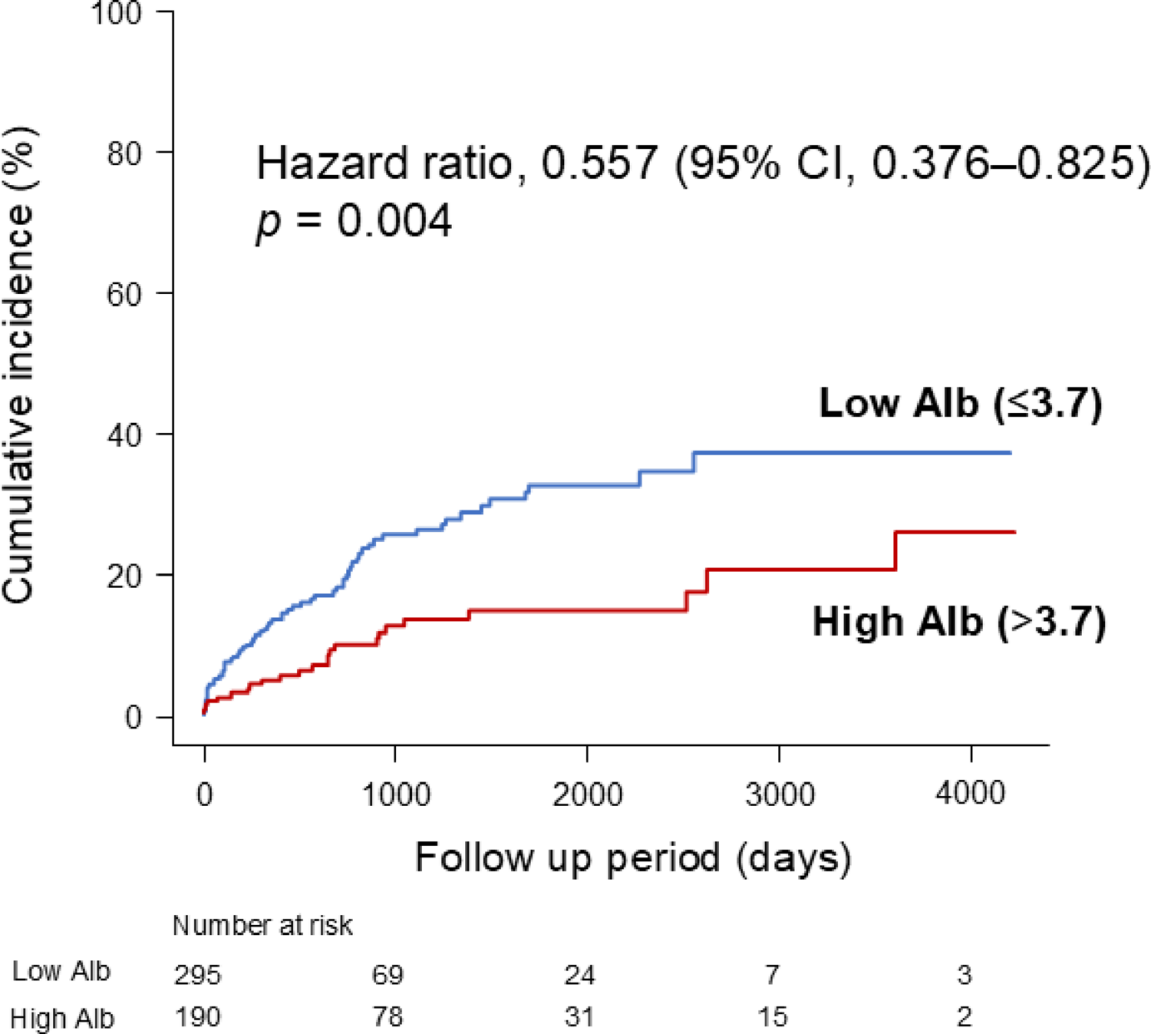
The cumulative incidence of cardiovascular death according to serum albumin levels after adjusting for the competing risks of death

To account for competing risks (e.g., cancer-related death), we used Fine-Gray sub-distribution hazard models to identify predictors of CV death. Univariate analysis revealed that age, albumin, eGFR, hemoglobin, and BNP levels were significantly associated with CV death (Table 3). The multivariate analysis demonstrated that patient age, higher BNP, and lower albumin levels were independent predictors of CV death after adjustment for confounding factors. Furthermore, we performed ROC analysis to evaluate the best cut-off value for predicting CV death in cancer survivors with HF. As shown in Fig. 3, the ROC analysis identified a serum albumin level of 3.7 g/dL as the threshold for predicting the incidence of CV death, with a sensitivity of 71.1%, a specificity of 46.2%, and an AUC of 0.588 (*p* < 0.001). When using the albumin cut-off value for CV death, the patients with low albumin levels had significantly higher risk for CV death than those with high albumin levels (Fig. 4).

**Table 3:** The Fine-Gray proportional hazard analyses for predicting the CV death

Variables	*Univariate analysis*			*Multivariate analysis*		
	HR	95% CI	*P* value	HR	95% CI	*P* value
Age^†^	1.456	1.160–1.833	0.001	1.443	1.108–1.882	0.007
Male gender	1.032	0.698–1.525	0.870			
BMI^†^	0.876	0.722–1.065	0.180			
Hypertension	1.270	0.815–1.979	0.290			
Diabetes mellitus	0.833	0.573–1.211	0.340			
Dyslipidemia	0.889	0.568–1.390	0.600			
Atrial fibrillation	1.097	0.751–1.602	0.630			
Prior hospitalization for HF	1.455	0.983–2.154	0.061			
LVEDD^†^	0.898	0.711–1.138	0.360			
LVEF^†^	0.843	0.692–1.035	0.088			
LAD^†^	1.035	0.836–1.287	0.730			
Albumin^†^	0.762	0.637–0.912	0.003	0.740	0.580–0.946	0.016
eGFR^†^	0.774	0.634–0.946	0.012	0.834	0.660–1.047	0.130
Hemoglobin^†^	0.813	0.677–0.975	0.026	0.954	0.741–1.228	0.710
Log BNP^†^	1.391	1.106–1.750	0.005	1.409	1.115–1.780	0.004

AF, atrial fibrillation; BMI, body mass index; BNP, B-type natriuretic peptide; HR, hazard ratio; CI, confidence interval; CV, cardiovascular; eGFR, estimated glomerular filtration rate; HF, heart failure; LAD, left atrial diameter; LVEDD, left ventricular end-diastolic diameter; LVEF, left ventricular ejection fraction. ^†^Per 1-SD increase.

## Discussion

### Main findings

The main findings of this study were as follows: (1) In cancer survivors with HF, CV death was the leading cause of mortality, occurring at a rate approximately four times higher than cancer-related death; (2) Low serum albumin levels were independently associated with an increased risk of CV mortality after adjusting for the competing risks of death such as cancer-related death; (3) A serum albumin cut-off value of 3.7 g/dL effectively stratified the patients by CV adverse events.

### Impact of serum albumin levels on clinical outcomes in cancer survivors with heart failure

It has been well known that cancer survivors are at increased risk for CV mortality compared to the general population, which is attributed to pre-existing CV risks and shared pathophysiological mechanisms such as chronic systemic inflammation and oxidative stress [11, 12]. On the other hand, the state of HF is associated with a higher risk for incident cancer [13]. Thus, since HF and cancer can mutually exacerbate each other, cancer survivors with coexisting HF are considered to have the highest CV event rates. Risk stratification for CV mortality plays a pivotal role in improving long-term clinical outcomes; however, little is known about the incidence and specific predictors for CV death in this high-risk group. In the present study, CV death was the most frequent cause of mortality, regardless of cancer types. Furthermore, our results demonstrated that serum albumin levels, apart from conventional predictors such as age and BNP, may serve as an independent and significant predictor for CV mortality among cancer survivors with HF. Hypoalbuminemia is well established as a marker of poor prognosis in both HF and cancer respectively, reflecting malnutrition, catabolic state, and chronic inflammation [7, 14, 15]. Although previous studies have independently shown that hypoalbuminemia is associated with adverse outcomes in patients with cancer and in those with HF, data evaluating its prognostic significance in cancer survivors who subsequently develop HF are scarce. Our current study extended this evidence by demonstrating that albumin can independently predict cardiovascular mortality among cancer survivors with HF even after accounting for the competing risks. The mechanisms by which hypoalbuminemia increased the risk of CV death are likely multifactorial. Lower albumin levels promote the extravasation of fluid into the interstitial space, exacerbating edema and volume overload. This increase in cardiac preload and afterload can contribute to worsening HF [16]. In addition, serum albumin levels decrease in response to systemic chronic inflammation and serve as a negative acute-phase reactant [17]. Albumin has antioxidant properties by scavenging reactive oxygen species, which helps reduce inflammation and atherosclerosis [18]. Persistent inflammation impairs cardiac function through the release of inflammatory cytokines, reducing myocardial contractility and subsequent increase in the risk of CV events [19]. These mechanisms are likely to interconnect and may further enhance CV vulnerability in cancer survivors.

Although hypoalbuminemia is defined as serum albumin levels ≤ 3.5 g/dL in patients with HF [20], our findings suggested that cut-off value of 3.7 g/dL for serum albumin levels could serve as a reliable biomarker to identify high-risk patients for CV mortality among cancer survivors with HF. Although the albumin cut-off value of 3.7 g/dL demonstrated reasonable sensitivity (71.1%), its specificity was modest (46.2%), and the AUC was relatively low (0.588). These findings indicate that while hypoalbuminemia is associated with increased CV mortality risk, serum albumin alone has limited discriminative ability for accurately identifying high-risk patients. The low AUC suggests that albumin should be considered a supportive rather than a standalone prognostic marker, reflecting its role as an integrative indicator of chronic inflammation and nutritional status. Therefore, this limitation highlights the need for multi-marker approaches that combine albumin with HF-specific biomarkers and clinical variables in cancer survivors with HF. Importantly, serum albumin is a routinely measured, low-cost biomarker available in most clinical settings. Cancer survivors with HF exhibiting hypoalbuminemia may warrant more rigorous evaluation and treatment for CV diseases and comprehensive interventions including nutritional support. Adding albumin to HF risk assessment in cancer survivors could contribute to earlier nutritional intervention and more aggressive optimization of guideline-directed medical therapy, potentially leading to improved clinical outcomes.

### Limitations

The current study had several limitations. First, this was a single-center study, and the generalizability of our findings to other populations may be limited. Second, a more detailed stratification by cancer types, stages, cardiotoxic cancer treatment, and diverse etiologies of HF could not be fully explored. Third, the cause of death was adjudicated based on medical records, which may introduce classification bias. Finally, the population was limited to cancer survivors who had completed primary treatment, and our results may not be generalizable to patients with active or metastatic disease. Further studies are needed to address these limitations.

## Conclusions

The present study revealed that CV mortality is a predominant cause of death in cancer survivors with HF. Low serum albumin levels were an independent predictor of CV mortality in this vulnerable patient population. These findings emphasize the importance of assessing serum albumin levels for risk stratification and may contribute to the development of intensive CV management among cancer survivors with HF.

## Data Availability

The data that support the findings of this study are available from the corresponding author upon reasonable request.
